# Efficacy of Prenatal Yoga in the Treatment of Depression and Anxiety during Pregnancy: A Systematic Review and Meta-Analysis

**DOI:** 10.3390/ijerph19095368

**Published:** 2022-04-28

**Authors:** I-Hui Lin, Chueh-Yi Huang, Shih-Hsiang Chou, Chia-Lung Shih

**Affiliations:** 1 Department of Physical Medicine and Rehabilitation, Ditmanson Medical Foundation Chia-Yi Christian Hospital, Chia-Yi City 600, Taiwan; 07289@cych.org.tw; 2 Department of Obstetrics and Gynecology, Ditmanson Medical Foundation Chia-Yi Christian Hospital, Chia-Yi City 600, Taiwan; 07695@cych.org.tw; 3 Department of Orthopedics, Kaohsiung Medical University Hospital, Kaohsiung Medical University, Kaohsiung 807, Taiwan; 4 Clinical Research Center, Ditmanson Medical Foundation Chia-Yi Christian Hospital, Chia-Yi City 600, Taiwan

**Keywords:** anxiety, depression, pregnancy, yoga

## Abstract

Women commonly suffer from depression during pregnancy. For reducing depression, yoga seems to be more suitable for pregnant women than other physical activities because of its low exercise intensity. The objective of this study was to assess the efficacy of prenatal yoga on the treatment of depression during pregnancy. Three electronic databases were searched for relevant articles from their inception to May 2021, including PubMed, Cochrane Library, and ScienceDirect. Pre- and post-test outcomes were adopted to estimate standardized mean difference with a 95% confidence interval for assessing the efficacy of yoga. Heterogeneity among articles was detected using *I*^2^ value. A total of 13 articles that contained 379 subjects were included for meta-analysis. No significant improvement in depression scores after practicing yoga was observed for women without depression (*p* = 0.09) but significant improvement was observed for women with depression (*p* = 0.001). Although significant improvement in anxiety scores after yoga was observed for women without depression (*p* = 0.02), the results of the sensitivity analysis were not consistent, while significant improvement in anxiety scores after yoga was also observed for women with depression (*p* < 0.00001). The current evidence has suggested that yoga had significant improvement in depression and anxiety scores in pregnant women with depression. However, the level of evidence of this study was not high. More articles with high levels of evidence should be conducted to confirm our conclusion in the future.

## 1. Introduction

Psychological disorders are commonly observed during pregnancy, and are also serious health problems, such as depression and anxiety [1,2]. Prenatal depression is even more prevalent than postpartum depression [3]. About 12% of women suffer from depression during pregnancy worldwide [4]. During pregnancy, several factors may result in these psychological disorders, such as physical or hormonal changes, and anxiety toward labor or fetal outcome [5]. Moreover, depression during pregnancy has been reported to be the cause of intrauterine fetal growth restriction, preterm delivery, or low birth weight [3,6]. Unfortunately, most women with perinatal depression appear unlikely to accept therapy [7].

Pharmacotherapy is the most common treatment for patients with depression, although most antidepressants have been shown to produce adverse fetal effects in animals [8]. One previous meta-analysis reported that the efficacy of antidepressants in the treatment of pregnant women with depression is still unclear [8]. These antidepressants may have adverse effects on the fetus, so non-pharmacological treatments seem to be a safer method for treating depression during pregnancy, such as psychotherapy, music therapy, and exercises [9,10,11]. 

Yoga is becoming a popular exercise worldwide. Yoga that combines postures, breathing and meditation is associated with the development of health and body awareness [12]. The benefits of yoga for humans have been validated by the scientific literature; for example, yoga can promote health and has therapeutic effects on disease, depression, stress, and anxiety [13,14,15]. Yoga seems to be more suitable for pregnant women than other physical activity due to its low exercise intensity [16]. A meta-analysis has been conducted to investigate the efficacy of yoga-based interventions for depression during pregnancy [17], and although this meta-analysis included articles with high levels of evidence (randomized controlled trials), these control groups included varying treatments, and thus the efficacy of yoga for treating depression during pregnancy is still unclear [17]. Recently, a meta-analysis has demonstrated the efficacy of yoga for depression during pregnancy by comparing pre–post changes [18]. Among those participants, however, some were depressed, while some were not depressed. Whether yoga is effective for women who are depressed during pregnancy is still undetermined. In addition, anxiety is a common disorder for pregnant women and 18.2% of women experience this disorder [19]. Depression is commonly accompanied by anxiety. However, previous meta-analysis articles did not consider the efficacy of yoga on anxiety for women with depression during pregnancy [17]. Thus, the efficacy of yoga on anxiety for pregnant women should be investigated.

This study aimed to conduct a meta-analysis for assessing the efficacy of prenatal yoga in the treatment of depression during pregnancy. Whether prenatal yoga was effective for pregnant women with depression was further investigated. These results could provide valuable clinical information regarding whether prenatal yoga was effective for pregnant women with depression and without depression, respectively. Psychiatrists could advise whether pregnant women with depression can receive yoga therapy based on our results.

## 2. Methods

### 2.1. Literature Search Process

This study was reported according to the Preferred Reporting Items for Systematic Reviews and Meta-Analyses 2020 statement [20], and the protocol was not registered and not prepared. Relevant articles that investigated the effect of yoga in the treatment of depression during pregnancy were collected in this study. Three electronic databases were searched for relevant articles from their inception to May 2021, including PubMed, Cochrane Library, and ScienceDirect. Five major topic terms, “yoga” AND “pregnancy OR pregnant” AND “depression OR anxiety”, were adopted to search relevant articles. The detailed search strategy for the three databases is demonstrated in Table S1. After the relevant articles were identified from these databases, the searching process was independently conducted by two authors (S.-H.C. and C.-L.S.). Firstly, the duplicates among these identified articles were highlighted and removed by using EndNote X8. The remaining articles were searched for relevancy by title/abstract screening, and then finally, the relevant articles could be confirmed by full-text screening; additionally, the reference lists of relative review articles were also searched for relevancy. Any disagreement was resolved by discussion until a consensus was reached.

### 2.2. Inclusion/Exclusion Criteria

The relevant articles meeting our inclusion criteria were included in this study. The inclusion criteria were: (1) subjects consisting of pregnant women who were depressed or not depressed; (2) subjects receiving yoga-based interventions; (3) presence of pre- and post-test designs; (4) available outcomes regarding depression and anxiety; and (5) articles being published in English or Chinese. The exclusion criteria for articles were: (1) conference abstract, note, letter, and review articles; (2) not only treated with yoga; (3) incomplete data for conducting meta-analysis; and (4) no clinical outcomes regarding depression or anxiety.

### 2.3. Data Extraction

A data extraction sheet was made by us. Data were independently extracted by the two authors. The extraction data included first author, publication year, country, the number of subjects, maternal age, gestational age, duration of yoga process, and outcomes. If the included articles did not provide detailed information, this was requested by correspondence with the authors via e-mail. Any disagreement was rechecked until a consensus was reached.

### 2.4. Statistical Analyses

All the analyses were conducted by using Review Manager Version 5.4 (Cochrane Collaboration, Oxford, England). All outcomes were continuous data expressed as mean, standard deviation (SD), and total number of subjects. However, the measures to assess depression or anxiety varied among articles, so standardized mean difference (SMD) with a 95% confidence interval (CI) was adopted to compare the difference between pre- and post-tests. A forest plot was used to demonstrate SDM and 95%CI for each article and pooled result. The participants were sub-grouped to those with and without depression, then subgroup analyses were conducted and the difference between subgroups was estimated. Two models were used to estimate SMD or MD according to heterogeneity among articles. Heterogeneity was detected using *I*^2^ value, and if the *I*^2^ value was larger than 50%, the articles showed heterogeneity and a random-effect model was adopted; otherwise, a fixed-effect model was adopted. Sensitivity analysis was performed to explore the impact of excluding one article at a time on results.

## 3. Results

### 3.1. Literature Search

**Figure 1:**
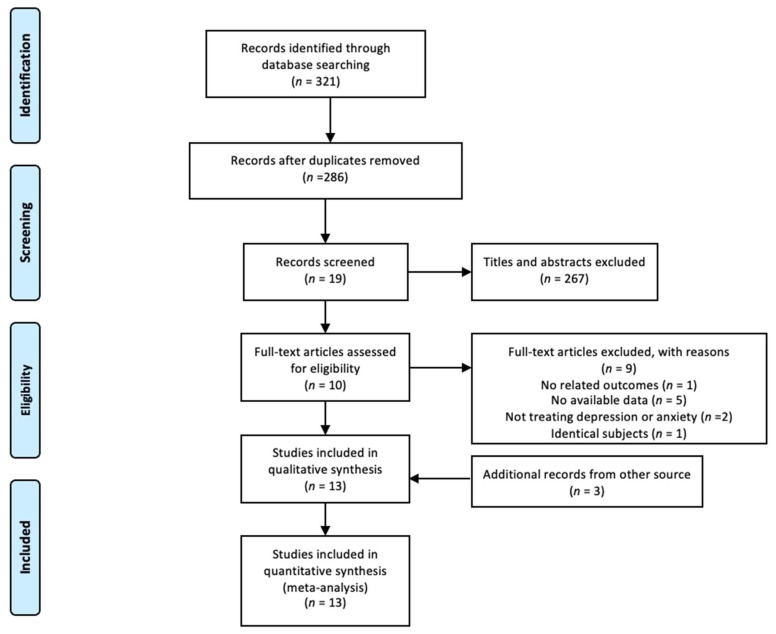
Flow diagram of searching process.

The results of the search strategy are shown in Figure 1. A total of 321 records were identified from the three databases. After removing duplicates, 286 records were further screened for relevant articles. Nineteen records were retained after title/abstract screening. Ultimately, 10 records met the eligibility criteria after full-text analysis, and an additional 3 records were identified from a review article. A total of 13 articles were included in our meta-analysis [16,21,22,23,24,25,26,27,28,29,30,31,32].

### 3.2. Major Characteristics of Included Articles

Major characteristics of the included articles are shown in Table S2, with most of the included articles (*n* = 6) being conducted in the USA, while other articles were conducted in various countries (two in Indonesia, two in Iran, one in India, one in Japan, and one in China). All of the 13 included articles contained 379 subjects. Beddoe 2009 group1 and group 2 were from the same article, in which group 1 was the subjects during the second trimester of pregnancy and group 2 was during the third trimester. These subjects’ mean maternal age ranged from 23.4 to 34.4 years, and their mean gestational age ranged from 19.9 to 27.6 weeks. Among these articles, six articles included pregnant women with depression, seven included pregnant women without depression, and one included pregnant women who were depressed or not depressed. Several self-reported measures (CES-D: Center for Epidemiological Studies Depression Scale, POMS: Profile of Mood States, HDS: Hamilton Depression Scale, EPDS: Edinburgh Postnatal Depression Scale, and HDRS: Hamilton Depression Rating Scale for assessing depression; STAI: State Anxiety Inventory, PASS: Perinatal Anxiety Screening Scale, SAS: Self-rating Anxiety Scale, HARS: Hamilton Anxiety Rating Scale, and PRAQ-R: Pregnancy-related Anxiety Inventory for assessing anxiety) were used to assess the severity of depression or anxiety.

### 3.3. Meta-Analysis

**Figure 2:**
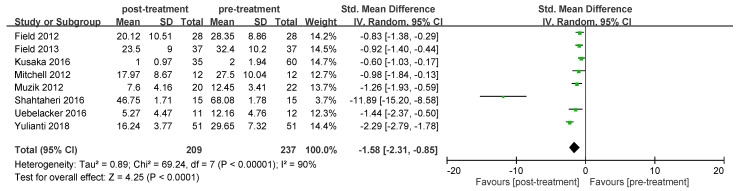
Forest plot of prenatal yoga practice in the treatment of depression for pregnant women with or without depression.

A total of nine articles reported self-reported scores to assess severity of depression. The pre- and post-treatment results of these articles were analyzed. The result demonstrated heterogeneity among these articles (*I*^2^ *=* 90%) and a random-effect model was used to compare the efficacy of yoga. After receiving prenatal yoga, pregnant women had significant improvement in depression compared with the pre-treatment level (random-effects model: 8 trials, SMD = −1.58, 95% CI = −2.31 to −0.85, *p* < 0.0001) (Figure 2). After conducting a sensitivity analysis, the results also demonstrated significant improvement in depression after treatment (Table S3).

**Figure 3:**
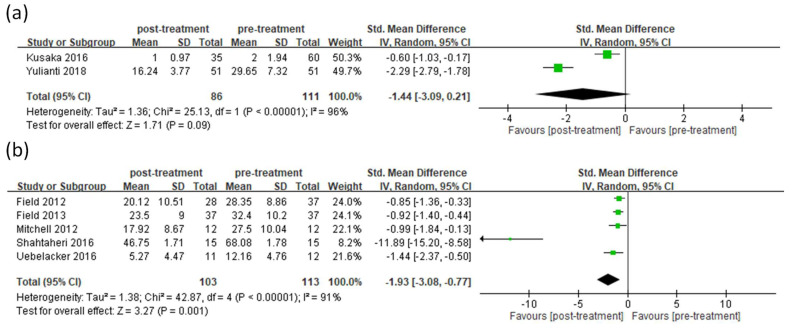
Forest plot of prenatal yoga in the treatment of depression for pregnant women without (**a**) or with depression (**b**).

Among the eight articles, two of them included pregnant women without depression. These two showed a heterogeneity (*I*^2^ *=* 96%), and a random-effect model was used to compare the effect of yoga. The result demonstrated that no significant improvement in depression after receiving prenatal yoga was observed for women without depression (random-effects model: 2 trials, SMD = −1.44, 95% CI = −3.09 to 0.21, *p* = 0.09) (Figure 3a). Five articles included pregnant women with depression. These articles showed a heterogeneity (*I*^2^ *=* 91%), and a random-effect model was adopted for meta-analysis. The result showed that significant improvement in depression after receiving prenatal yoga was observed for women with depression (random-effects model: 5 trials, SMD = −1.93, 95% CI = −3.08 to −0.77, *p* = 0.001) (Figure 3b). After conducting a sensitivity analysis, the results also demonstrated significant improvement in depression after treatment (Table S3).

**Figure 4:**
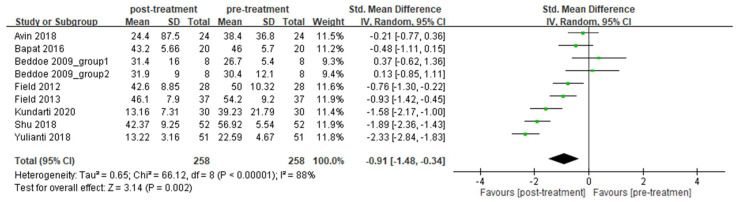
Forest plot of prenatal yoga in the treatment of anxiety for pregnant women with or without depression.

**Figure 5:**
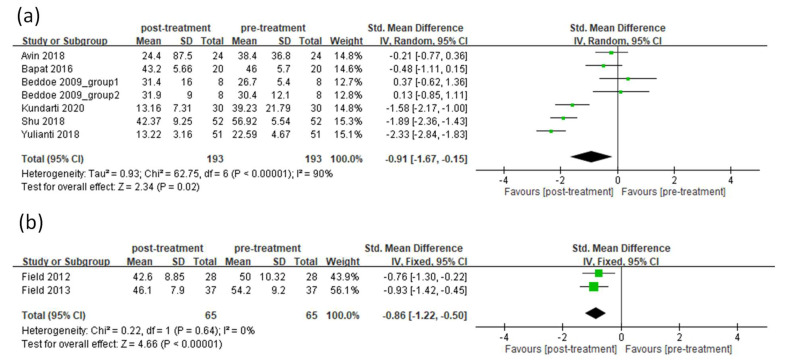
Forest plot of prenatal yoga in the treatment of anxiety for pregnant women without (**a**) or with depression (**b**).

A total of eight articles reported self-reported scores to assess severity of anxiety. Heterogeneity was detected among these articles (*I*^2^ *=* 88%), and a random-effect model was adopted for meta-analysis. After receiving prenatal yoga therapy, pregnant women had significant improvement in anxiety compared with the pre-treatment level (random-effects model: 9 trials, SMD = −0.91, 95% CI = −1.48 to −0.34, *p* = 0.002) (Figure 4). After conducting a sensitivity analysis, the results also demonstrated significant improvement in anxiety after treatment (Table S4). Among the eight articles, six of them included pregnant women without depression. These articles demonstrated heterogeneity (*I*^2^ = 90%), and a random-effects model was adopted for meta-analysis. The result demonstrated that significant improvement in anxiety after receiving prenatal yoga was observed for women without depression (random-effects model: 7 trials, SMD = −0.91, 95% CI = −1.67 to −0.15, *p* = 0.02) (Figure 5a). After conducting a sensitivity analysis, the results were not consistent (Table S4). Two articles included pregnant women with depression, but these did not demonstrate heterogeneity (*I*^2^ = 0%), and a fixed-effects model was adopted for meta-analysis. The result demonstrated that significant improvement in anxiety after receiving prenatal yoga was observed for women with depression (fixed-effects model: 2 trials, SMD = −0.86, 95% CI = −1.22 to −0.50, *p* < 0.00001) (Figure 5b). After conducting a sensitivity analysis, the results also demonstrated significant improvement in anxiety after treatment (Table S4).

### 3.4. Adverse Effects

Only one article assessed the adverse effects for each participant after receiving yoga, and no adverse effects were observed [28].

## 4. Discussion

### 4.1. Summary of Results

This study aimed to assess the efficacy of prenatal yoga in the treatment of depression during pregnancy. The results demonstrated that pregnant women with or without depression receiving prenatal yoga had significant improvement in depression even after conducting a sensitivity analysis. For subgroup analyses, significant improvement in depression scores after yoga was observed for pregnant women with depression, but no significant improvement was observed for those without depression. Pregnant women with or without depression receiving yoga therapy had significant improvement in anxiety even after conducting a sensitivity analysis. For subgroup analyses, significant improvement in anxiety was also observed for pregnant women with depression. Although significant improvement in anxiety was observed for women without depression, the results of its sensitivity analysis were not consistent.

### 4.2. Yoga on Depression

Although the previous articles have conducted meta-analysis including randomized controlled trials to assess the efficacy of prenatal yoga for treating depression during pregnancy [17,33], the control groups in these trials were administered various different therapies, so their results regarding the efficacy of prenatal yoga should be further confirmed. To avoid the effect of the control groups being administered various therapies, we adopted pre- and post-test results to assess the efficacy of prenatal yoga. Our results demonstrated that a significant improvement in depression was observed for pregnant women with or without depression, and our sensitivity analysis also supported that prenatal yoga was effective for depression during pregnancy. A previous meta-analysis which adopted pre- and post-test outcomes also showed similar results [18]. However, they did not consider if yoga is effective for pregnant women without depression.

A previous meta-analysis conducted subgroup analysis and concluded that prenatal yoga was effective not only for pregnant women with depression but also for those without depression [18]. However, it included randomized controlled trials, but these control groups contained various therapies. In this study, we attempted to investigate the efficacy of prenatal yoga on depression for pregnant women with and without depression, respectively. Our results demonstrated that prenatal yoga was effective for pregnant women with depression, but not effective for those without depression. Our results seem to be logical, as although pregnant women are not depressed, they might not obtain improvement in depression scores after receiving prenatal yoga.

### 4.3. Yoga on Anxiety

Depression is commonly accompanied by anxiety and, accordingly, the efficacy of prenatal yoga on anxiety for pregnant women was also investigated, with the results showing that yoga could significantly decrease anxiety for this group, with or without depression. This effect was also observed for pregnant women with depression; however, the effect of yoga on anxiety for pregnant women without depression was unclear because the results from the sensitivity analysis were not consistent. We classified pregnant women into depression and non-depression groups. However, some pregnant women without depression may be suffering from anxiety. This may explain why the effect of yoga on anxiety for pregnant women without depression was unclear. These results were similar to those derived from depression scores. It could be concluded that these results regarding the efficacy of yoga in the treatment of depression for pregnant women with depression appear reliable.

Among all the meta-analyses (Figure 2, Figure 3, Figure 4 and Figure 5), all of them demonstrated heterogeneity except for the sub-group analysis (anxiety for pregnant women without depression, Figure 5b). The *I*^2^ value of this sub-group analysis was zero. The sub-group analysis included two articles, and these articles were conducted by the same first author. This may indicate that the processes of experiments among these articles were similar, and the outcomes obtained from these articles should be similar. This may explain why the *I*^2^ value of this subgroup analysis was zero.

### 4.4. Safety of Yoga

Among our included articles, only one assessed the adverse effects after receiving yoga therapy and no adverse effects were observed [28]. A previous meta-analysis investigated the safety regarding yoga, and the results showed that injuries or adverse effects related to yoga are rare [34]. The poses and exercises of yoga could be adjusted to fit the situation of pregnant women and the risk of injuries could be decreased [35].

### 4.5. Limitation

There are some limitations in this study. Firstly, various self-reported measures were adopted to assess the severity of depression or anxiety, so these various measures might be the cause of heterogeneity among articles; moreover, some measures (CES-D and SCID) might not be the best method for assessing depression among pregnant women because some items (such as tiredness and lack of energy) could be misinterpreted as regular symptoms of pregnancy [17]. Secondly, the types of yoga in the included articles also varied, again possibly resulting in different effects on depression, thereby producing heterogeneity in the results. Thirdly, a limited number of trials were adopted in some subgroup analyses and a sensitivity analysis could not be performed. Fourthly, our results demonstrated a significant decrease in depression levels for pregnant women with depression after yoga, but whether they became not depressed after yoga was still unknown. Fifthly, the included articles were limited to those published in English or Chinese. Some articles published in other languages might have been missed. Finally, our meta-analysis adopted pre- and post-test results and the current results had a low level of evidence. More articles with high-level evidence (such as randomized controlled trials) using a consistent control group should be further conducted to assess the efficacy of prenatal yoga in the treatment of depression during pregnancy.

## 5. Conclusions

To clarify the efficacy of prenatal yoga in the treatment of depression during pregnancy, we conducted a meta-analysis to assess the efficacy of yoga. The current evidence has suggested that yoga was effective in reducing depression and anxiety for pregnant women with or without depression. For subgroup analyses, our results showed that yoga was effective in reducing depression and anxiety for pregnant women with depression, but it was not effective for those without depression. We advised that pregnant women with depression should seek professional help. Among several therapies, yoga is a safe and effective treatment for pregnant women with depression based on our results. The patients should discuss with their psychiatrist whether yoga is optimal therapy for their condition. However, our results lacked a high level of evidence, and more randomized controlled trials should be further conducted to confirm our results.

## Data Availability

All data were available from our included articles.

## Supplementary Materials

The following supporting information can be downloaded at: https://www.mdpi.com/article/10.3390/ijerph19095368/s1. Table S1 is the detail of search strategy. Table S2 is the major characteristics of included articles. Table S3 is sensitivity analysis for the effect of yoga on changes in depression scores. Table S4 is sensitivity analysis for the effect of yoga on changes in anxiety scores.
