# Supplementary material for: Efficacy of Prenatal Yoga in the Treatment of Depression and Anxiety during Pregnancy: A Systematic Review and Meta-Analysis
Source: Int J Environ Res Public Health. 2022 Apr 28;19(9):5368. doi: 10.3390/ijerph19095368 (PMC9105819; doi:10.3390/ijerph19095368)
Supplement: Supplementary file 1 [file ijerph-19-05368-s001.zip › ijerph-1614614-supplementary.pdf]

Table S1. Search strategy.

Database	Search details
PubMed	(yoga) AND ((pregnancy) OR (pregnant)) AND ((depression) OR (anxiety)) “Title/Abstract“
Cochrane Library	(yoga) AND ((pregnancy) OR (pregnant)) AND ((depression) OR (anxiety)) “Title/Abstract/Keyword“
ScienceDirect	(yoga) AND ((pregnancy) OR (pregnant)) AND ((depression) OR (anxiety)) “Title/Abstract/Author-specified keywords“

Table S2. Major characteristics of included articles.

Authors	Country	Participant with depression	Total number of subjects	Maternal age (year)	Gestational age (week)	Treatment duration (week)	Extracted outcome
Beddoe 2009_group1	USA	No	8	30.4	13-26	7	STAI
Beddoe 2009_group2		No	8		27-32	7	
Field 2012	USA	Yes	28	NA	20	12	STAI, and CES-D
Mitchell 2012	USA	Yes	12	NA	NA	12	CES-D
Muzik 2012	USA	Both	22	32.41±4.98	21.8±5.96	10	EPDS
Field 2013	USA	Yes	37	24.4±4.7	NA	12	STAI, and CES-D
Bapat 2016	India	No	20	NA	NA	4	STAI
Kusaka 2016	Japan	No	60	34.4±4.1	19.9±1.4	15	POMS
Shahtaheri 2016	Iran	Yes	15	NA	NA	8	HDS
Uebelacker 2016	USA	Yes	12	28.0±5.9	NA	9	EPDS
Avin 2018	Iran	No	24	24.2±1.8	NA	4~5	PRAQ-R
Shu 2018	China	No	52	29.29±4.80	NA	4	SAS
Yulianti 2018	Indonesia	No	51	26.65±4.04	27.57±4.98	4	HARS and HDRS
Kundarti 2020	Indonesia	No	30	23.4±3.27	23.5±2.83	8	PASS

NA: not available. STAI: State Anxiety Inventory. CES-D: Center for Epidemiological Studies Depression Scale. EPDS: Edinburgh Postnatal Depression Scale. POMS: Profile of Mood States. HDS: Hamilton Depression Scale. PRAQ-R: Pregnancy-related Anxiety Inventory. SAS: Self-rating Anxiety Scale. HARS: Hamilton Anxiety Rating Scale. HDRS: Hamilton Depression Rating Scale. PASS: Perinatal Anxiety Screening Scale.

Table S3. Sensitivity analysis for the effect of yoga on changes in depression scores.

Altered dataset	Model	SMD	95% CI	p-value	I^2
**Women with or without depression**					
Removal of Field 2012	random	-1.76	(-2.61, -0.90)	<0.0001	91
Removal of Field 2013	random	-1.77	(-2.65, -0.89)	<0.0001	91
Removal of Kusaka 2016	random	-1.80	(-2.64, -0.95)	<0.0001	90
Removal of Mitchell 2012	random	-1.70	(-2.51, -0.88)	<0.0001	91
Removal of Muzik 2012	random	-1.68	(-2.52, -0.84)	<0.0001	91
Removal of Shahtaheri 2016	random	-1.18	(-1.67, -0.69)	<0.00001	79
Removal of Uebelacker 2016	random	-1.62	(-2.43, -0.82)	<0.0001	91
Removal of Yulianti 2018	random	-1.41	(-2.13, -0.68)	0.0002	87
**Women with depression**					
Removal of Field 2012	random	-2.69	(-4.45, -0.92)	0.003	93
Removal of Field 2013	random	-2.70	(-4.50, -0.90)	0.003	93
Removal of Mitchell 2012	random	-2.38	(-3.85, -0.90)	0.002	93
Removal of Shahtaheri 2016	fixed	-0.96	(-1.26, -0.65)	<0.00001	0
Removal of Uebelacker 2016	random	-1.00	(-1.33, -0.68)	<0.00001	93

SMD: standardized mean difference. CI: confidence interval.

Table S4. Sensitivity analysis for the effect of yoga on changes in anxiety scores.

Altered dataset	Model	SMD	95% CI	p-value	I^2
**Women with or without depression**					
Removal of Avin 2018	random	-1.01	(-1.60, -0.42)	<0.0001	87
Removal of Bapat 2016	random	-0.96	(-1.58, -0.34)	0.002	89
Removal of Beddoe 2009	random	-1.18	(-1.76, -0.60)	<0.00001	88
Removal of Field 2012	random	-0.96	(-1.57, -0.28)	0.005	89
Removal of Field 2013	random	-0.90	(-1.56, -0.24)	0.008	89
Removal of Kundarti 2020	random	-0.82	(-1.45, -0.19)	0.01	89
Removal of Shu 2018	random	-0.78	(-1.38, -0.18)	0.01	87
Removal of Yulianti 2018	random	-0.74	(-1.26, -0.22)	0.006	83
**Women without depression**					
Removal of Avin 2018	random	-1.04	(-1.84, -0.24)	0.01	89
Removal of Bapat 2016	random	-0.98	(-1.84, -0.13)	0.02	91
Removal of Beddoe 2009	random	-1.31	(-2.11, -0.51)	0.001	91
Removal of Kundarti 2020	random	-0.79	(-1.69, 0.12)	0.09	92
Removal of Shu 2018	random	-0.73	(-1.61, 0.51)	0.1	91
Removal of Yulianti 2018	random	-0.67	(-1.43, 0.09)	0.08	88

SMD: standardized mean difference. CI: confidence interval.
